# Draft genome of halophilic *Salinicoccus roseus* H15 isolated from desert rock varnish in Ma'an, Jordan

**DOI:** 10.1128/mra.00928-24

**Published:** 2024-12-12

**Authors:** Sulaiman M. Alnaimat, Saqr Abushattal, Saif M. Dmour

**Affiliations:** 1 Department of Medical Analysis, Princess Aisha Bint Al-Hussein College of Nursing and Health Sciences, Al-Hussein Bin Talal University, Ma’an, Jordan; University of Southern California, Los Angeles, California, USA

**Keywords:** *Salinicoccus roseus*, desert rock varnish, Ma'an, Jordan, halophilic

## Abstract

Here, we report the draft genome sequence of *Salinicoccus roseus* H15, isolated from desert rock varnish in Ma'an, Jordan. The genome size is 2,457,773 bp with a GC content of 49.31 mol%. The genome includes 3,064 genes and 135 carbohydrate-active enzymes, demonstrating adaptations for survival in extreme environments.

## ANNOUNCEMENT

The genus *Salinicoccus*, first described by Ventosa et al. in 1990 (1), consists of moderately halophilic, aerobic, Gram-positive cocci (1). According to the LPSN, the genus currently includes 18 validly published species (2). Among these, *Salinicoccus roseus* strains have demonstrated significant industrial potential, showing high sun protection factor and lignin peroxidase activity, which are valuable for bioremediation and eco-friendly industries (3, 4).

**Fig 1:**
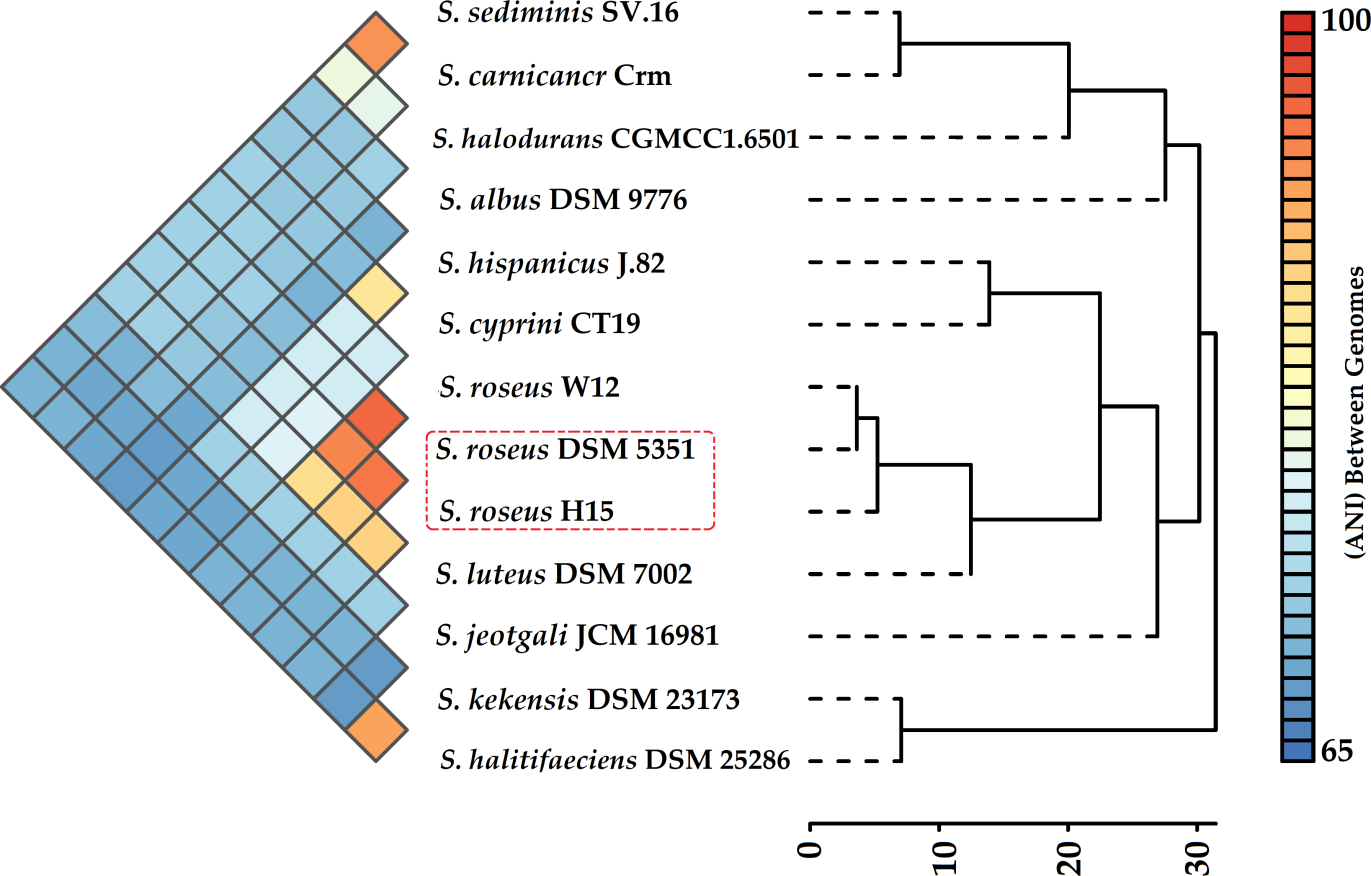
Heatmap and hierarchical clustering based on pairwise average nucleotide identity (ANI) values of *Salinicoccus roseus H15* and the reference species of the genus *Salinicoccus* and the closest related strain. The analysis was performed using the Integrated Pan-Genome Analyser (https://nmdc.cn/ipga/) (13). The heatmap illustrates the ANI values, with the color gradient representing the percentage identity, ranging from 65% to 100%. The dendrogram on the right shows the hierarchical clustering, indicating the evolutionary relationships among the strains. The scale under the dendrogram, ranging from 0 to 30, reflects the distance based on ANI, with higher values indicating greater dissimilarity between strains.

On 11 November 2014, desert rock varnish samples were aseptically collected from a semiarid region near Ma'an, Jordan (30.188836, 35.639121), characterized by approximately 50 mm of annual rainfall (5). Flat rocks were selected, sealed in sterile foil, and transported to the lab under sterile conditions. The rock surfaces were ground into varnish powder using a flame-sterilized coarse bit, and the powder was stored at −4°C. A 0.1 g sample of the powdered rock varnish was inoculated onto nutrient agar (Oxoid), supplemented with 2M NaCl (117 g/L). After 72 hours of incubation at 37°C, distinct yellow to orange, smooth, circular colonies (2–3 mm in diameter) were isolated and streaked twice for purification. Genomic DNA was directly extracted from the colony of interest using the G-spin Total DNA Extraction Mini Kit (iNtRON Biotechnology, Korea), and the purity and integrity were evaluated using a Nabi-UV/Vis Nano Spectrophotometer (MicroDigital, South Korea). Sequencing libraries were prepared using the Illumina DNA Prep tagmentation kit and sequenced on the Illumina NextSeq 2000 platform at EzBiome Inc. (Gaithersburg, MD, USA), generating approximately 15 million paired-end reads (150 bp). Read quality was assessed using FastQC v0.11.9 (6), and MultiQC v1.11 (7) was used for report generation. Genome assembly was performed with SPAdes v3.15.5 (8), resulting in 148.96× coverage across 2,481,724 sequences with an average read length of 147.52 bp, followed by the identification and removal of contaminant sequences using the NCBI Foreign Contamination Screen (FCS) (9). Genome completeness and contamination were further assessed using CheckM v1.1.2 (10), which revealed 99.14% completeness and 1.72% contamination. Genome quality assessment using QUAST v4.4 (11) indicated 43 contigs ≥500 bp (from a total of 573 publicly available contigs), with the largest contig measuring 366,482 bp. The total genome length was 2,457,773 bp, featuring an N50 value of 181,087 bp and a GC content of 49.31%. Statistics were based on contigs ≥500 bp, and default software parameters were used unless specified. Taxonomic validation, including average nucleotide identity (ANI), was conducted using GTDB-Tk v1.7.0 via Kbase (12), confirming the organism as *Salinicoccus roseus* DSM 5351 (GCF_003814515.1) with a FastANI value of 95.88%, as visualized in the pairwise ANI-based heatmap (Fig. 1) generated with integrated pan-genome analyser (IPGA) (13). The draft genome was annotated using (PGAP) v6 (14). The annotation process identified 3,064 total genes, 2,992 CDSs, of which 2,946 were coding genes, and 2,946 CDSs contained proteins. Additionally, 72 RNA genes were identified, including 11 rRNAs including rRNAs (2, 2, 7 for 5S, 16S, and 23S), and 57 tRNAs. There were also four ncRNAs. Forty-six pseudogenes were identified. The Protologger (15) functional analysis identified 149 transporters, 22 secretion genes, and 698 unique enzymes; no CRISPR arrays were found. CAZyme analysis found 135 carbohydrate-active enzymes, highlighting metabolic versatility.

## Data Availability

The annotated draft genome sequence has been deposited in the NCBI under the DDBJ/ENA/GenBank accession number JBGGGS000000000. The version described in this paper is version JBGGGS010000000. The BioProject database accession number is PRJNA1144690, and the BioSample database accession number is SAMN43042367. The Sequence Read Archive information is available under the accession number SRR30201806.
